# 16 Association of Frailty and Comorbidities with Burn Outcomes: A Multicenter Study

**DOI:** 10.1093/jbcr/irac012.020

**Published:** 2022-03-23

**Authors:** David L Wallace, Joyce E Wall, Angela Man, Jason Heard, Najib M Allabadi, Marc G Jeschke, Alisa Savetamal, John T Schulz, Kathleen S Skipton Romanowski

**Affiliations:** University of California, Davis, Sacramento, California; Bridgeport Hospital, Seymour, Connecticut; Massachusetts General Hospital, Boston, Massachusetts; University of California - Davis, Sacramento, California; Vohra physician group, Fresno, California; U of T, Toronto, Ontario; Yale New Haven System/Bridgeport Hospital, Bridgeport, Connecticut; Massachusetts General Hospital, Boston, Massachusetts; UC Davis, Sacramento, California

## Abstract

**Introduction:**

Previous work has demonstrated the association of increased frailty and mortality in burn patients, but the impact of specific co-morbidities and frailty on burn patients’ short term outcomes has not been explored. The purpose of this study was to determine the relationship of frailty and patient comorbidities on in-hospital mortality and length of stay (LOS).

**Methods:**

A retrospective chart review of all acutely injured burn patients admitted from January 2016 - December 2017 at 3 US ABA verified burn centers was conducted. Demographics and all comorbidities included in the burn database were collected. The modified frailty index-11 score (MFI) was calculated for each patient. Descriptive statistics, univariate and multivariate analysis were completed to determine the relationship between frailty and comorbidities with mortality, LOS, and LOS/% Total Body Surface Area (%TBSA).

**Results:**

1615 patients were included. Mean age was 45.9 + 17.7 years and 1145 (70.9%) were male. Mean %TBSA was 9.6%+14.2% and mean MFI was 0.43 + 0.74. The mean LOS was 12.3 days + 21.1. A total of 1542 (95.5%) patients survived to discharge. The most common co-morbidities present on admission were: smoking (336, 22.7%), hypertension (HTN, 313, 19.4%), drug dependence (247, 15.3), diabetes (DM, 175, 10.8%), alcoholism (171,10.6%), major psychiatric illness (MPI, 169,10.5%), heart failure (CHF, 23, 1.4%), obesity (7, 4.3%), and respiratory disease (RD, 136, 8.4%). Multivariate logistic regression revealed that RD (OR 3.6, 95%CI 1.4-9.4), age (OR 1.1, 95%CI 1.06-1.1), and %TBSA (OR 1.1, 95%CI 1.1-1.17) were independently predictive of mortality. Multiple linear regression demonstrated patients without alcoholism (β -3.9 95% CI -5.7- -2.1), MPI (β -3.8 95% CI -4.9- -3.0), drug dependence (β -3.9 95% CI -5.7- -2.1), and DM (β -2.0 95% CI -5.7- -2.8) had shorter LOS. Though MFI, heart failure, DM, MPI, alcoholism, and HTN, were all significant for LOS/%TBSA in univariate analysis, they were NOT significant in the multivariate linear regression model.

**Conclusions:**

MFI does not independently contribute to mortality or LOS when accounting for other patient co-morbidities. Respiratory disease on admission is associated with mortality, and major psychiatric illness and drug dependence increase LOS. This information will be used to develop interventions for these groups in order to improve mortality, and decrease LOS.

